# Documentation of antimicrobial indication and duration in electronic health records in a rehabilitation hospital: a cross-sectional study

**DOI:** 10.1017/ash.2025.10259

**Published:** 2025-12-19

**Authors:** Lilah Moccia, Cassidy Arvinte, Jennifer Lee, Bradley J. Langford

**Affiliations:** 1 Faculty of Health Sciences, McMaster University, Hamilton, ON, Canada; 2 American University of the Caribbean School of Medicine, Cupecoy, St. Maarten; 3 Hotel Dieu Shaver Health and Rehabilitation Centre, St. Catharines, ON, Canada; 4 Hotel Dieu Shaver Health and Rehabilitation Centre, McMaster University, Hamilton, ON, Canada; 5 Department of Health Research Methods, Evidence and Impact, McMaster University, Hamilton, ON, Canada

## Abstract

In this cross-sectional study of 136 antimicrobial prescriptions at a rehabilitation hospital with recent implementation of electronic medication order entry and no mandatory requirements, we found incomplete documentation of both indication (37%) and duration (75%). An accompanying survey identified potential solutions including reminders, improved choice architecture, and forced functions.

## Introduction

Appropriate antimicrobial prescribing can be facilitated through electronic medical records. Clinical decision support encompasses a range of supports to optimize antibiotic prescribing, including order sets for antibiotic selection, dose, and duration, positive culture alerts, and risk stratification for antimicrobial resistance. However, foundational to such efforts is appropriate documentation and order attributes when prescribing antimicrobials.

Indication and duration documentation on antimicrobial prescriptions are two such examples. Including an indication on an antimicrobial prescription is recommended to help facilitate selection of appropriate antimicrobial regimens, communication between the interprofessional team, and antimicrobial stewardship audits.^1,2^ Despite these purported benefits, antimicrobial indication is often not included as part of the prescription.^3,4^ Similarly, a lack of inclusion of duration on antimicrobial prescriptions risks unnecessarily prolonged duration and preventable harm for patients. However, there are limited data on how often duration is missing from prescriptions, and the potential impact of such omissions.

A better understanding of the current documentation practice and barriers and facilitators to optimal practice is needed, particularly in less studied settings like rehabilitation hospitals. This will help inform strategies to improve antibiotic stewardship and promote safer, more effective prescribing for our patients. Our objective was to evaluate the prevalence of antimicrobial indication and duration documentation and describe the determinants to improve documentation.

## Methods

### Study design

Cross-sectional study which includes both a retrospective chart review of antimicrobial prescriptions, and a survey of prescribers.

### Setting

Hotel Dieu Shaver Health and Rehabilitation Centre is a complex continuing care and rehabilitation facility in Ontario, Canada. As a community-based rehabilitation center, it services 137 beds broken into 37 acute rehabilitation beds allocating 2–3 hours a day to rehabilitation, 85 low intensity rehabilitation beds allocating 1–2 hours a day, 10 medical complex beds receiving medical services only and 5 palliative beds.

An electronic health record (EHR) has been newly launched in November of 2024 with computerized provider order entry (CPOE) used for all medication orders. The CPOE system allows the prescriber to select one of several indications via drop-down selection, or via free-text. The latter option may be used to indicate multiple indications or diagnostic uncertainty. Indication and duration were not mandatory fields in the EHR. Education for the providers on the new electronic health system was completed prior to go-live of the new system. This was a combination of electronic training modules, in person training, group education and self-directed learning.

### Participants

Patients with antimicrobial prescriptions during the period of January 2025 to June 2025 were included. All prescribers were eligible for participation in the survey.

### Outcomes

The primary outcome was the proportion of antimicrobial prescriptions with a 1) documented indication and 2) documented duration included in the prescription order.

We also investigated the barriers and facilitators to antimicrobial prescription documentation to inform future efforts.

### Data sources

Indication and duration documentation was collected from Cerner Millennium EHR. One author generated a list of consecutive patients on antimicrobials during the study period, and a second author reviewed the list and each patient’s order to determine the indication and duration attributes directly associated with the antimicrobial prescription.

To investigate the barriers and facilitators to appropriate antimicrobial prescription documentation, we launched a survey on June 20, 2025 which closed on August 16, 2025. The survey was distributed through a QR code that linked to a Microsoft Form for providers to complete. Survey outcomes include the perceptions surrounding indication and duration documentation, and barriers and facilitators to ensuring these attributes are documented on all prescriptions. The survey questions and responses are included in the Supplement.

### Analysis

We presented results descriptively, calculating the percentage of antimicrobial prescriptions with an indication or duration included as part of the prescription. A minimum sample of 81 was required to achieve 95% confidence and 10% precision assuming prevalence of indication is approximately 30% and duration is approximately 70%. Survey results were also presented descriptively.

### Ethics statement

This quality review was analyzed and approved by Hotel Dieu Shaver Health and Rehabilitation Centre Research Ethics Board.

## Results

### Documentation of indication and duration

**Figure 1.**
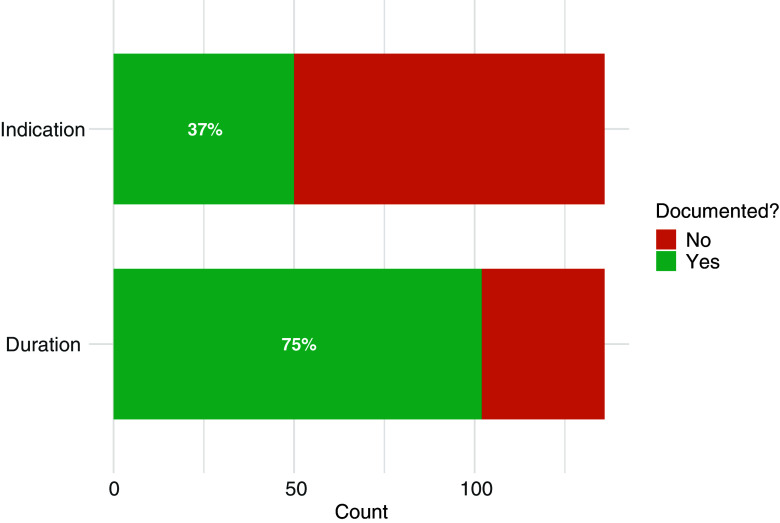
Antimicrobial indication and duration documentation in a rehabilitation hospital.

A total of 136 patient charts were reviewed and analyzed. Out of the 136 antimicrobial prescriptions, 50 (37%) included a documented indication. There were no instances of multiple indications or diagnostic uncertainty when indications were provided. Most common indications were urinary (*N* = 22, 44%), skin and soft tissue (*N* = 16, 32%), respiratory tract (*N* = 7, 14%), surgical (*N* = 3, 6%), and otolaryngologic (*N* = 2, 4%). Out of the same 136 prescriptions, 102 (75%) included a documented duration (Figure 1).

### Barriers and facilitators to documentation

A total of 11 provider surveys were received and analyzed out of 19 total prescribers approached, a 58% response rate. Responses were received from 3 nurse practitioners and 8 medical doctors. Half of respondents did not find the indication or duration features of the EHR order easily visible or accessible. Respondents indicated that system navigation (*n* = 5, 62%), time required (*n* = 4, 50%), and workflow related challenges, such as calling in orders from offsite (*n* = 3, 38%) were the main barriers to indication and duration documentation. Suggested improvements to improve documentation included reminders (eg, popup upon order entry), improved choice architecture (eg, check boxes, order sets with preset duration and indication) and forced functions (eg, making indication and duration mandatory). The results from all survey questions are displayed in Supplementary Tables A–F.

## Discussion

In this single center study of a rehabilitation hospital with recent implementation of electronic medication order entry and no mandatory requirements, we found incomplete documentation of both antimicrobial indication (37%) and duration (75%). These findings align with other research that has shown wide ranges in interfacility documentation,^4^ and particularly low rates of indication documentation in long-term care settings where electronic tools to facilitate complete documentation are less common.^3^

Inadequate documentation may be a key barrier to antimicrobial stewardship. A scoping review on indication documentation showed that 17 of 19 studies found an association between indication documentation and antimicrobial appropriateness.^4^ Indication documentation can facilitate inter disciplinary assessment of appropriateness and increase prescriber accountability. Duration documentation reduces the likelihood that antimicrobial prescriptions are continued for an unnecessarily long course.

Our survey responses indicated that the new EHR was difficult to navigate, and the system did not facilitate indication and duration documentation. There was limited awareness of the indication and duration fields within the electronic ordering system. This knowledge gap may have contributed to lower documentation rates, suggesting the importance of clinician engagement and education regarding new EHR functionality. Respondents suggested practical solutions such as reminders, order sets, and mandatory fields to ensure these aspects of the prescription were documented. Research supports the value of mandatory indication fields within EHR systems, which is shown to be feasible and result in accurate documentation.^5^ However, barriers such as time constraints and disrupted workflow may hinder such efforts.^6,4^

This study has several limitations that should be considered when analyzing the results. The small sample size limits our ability to generalize findings and may not fully capture the accuracy of prescribing behaviors across other hospitals. Our study reviewed patient medical records over a four month period during spring 2025. The short interval of time may not reflect broader prescribing trends and restrict the ability to observe fluctuations in antibiotic use that arise from seasonal variations, staffing changes, or evolving stewardship efforts. This study focused solely on the presence or absence of indication and duration documentation and did not evaluate the accuracy of clinical indication or appropriateness of the selected indications. Future research efforts could cross-reference provider-selected indications with clinical documentation to improve the assessment of appropriateness.

Nevertheless, this study focuses on a rehabilitation hospital, a less common and less resourced setting from an antimicrobial stewardship perspective. Findings from this research will address identified barriers and facilitators so that the antibiotic stewardship team can devise strategies to improve antibiotic documentation.

## Conclusion

There are significant gaps in the documentation of antibiotic indication and duration in the context of a new EHR lacking mandatory requirements for these fields. We identified challenges in EHR utilization among providers, including limited awareness and difficulty navigating indication and duration fields. These findings emphasize the need for targeted interventions, including mandatory documentation fields, order sets, and clinical decision support tools to strengthen antibiotic stewardship and facilitate effective communication in hospital settings.

## Supporting information

10.1017/ash.2025.10259.sm001 Moccia et al. supplementary material Moccia et al. supplementary material

## Supplementary material

The supplementary material for this article can be found at https://doi.org/10.1017/ash.2025.10259
