# Supplementary material for: Documentation of antimicrobial indication and duration in electronic health records in a rehabilitation hospital: a cross-sectional study
Source: Antimicrob Steward Healthc Epidemiol. 2025 Dec 19;6(1):e4. doi: 10.1017/ash.2025.10259 (PMC12766525; doi:10.1017/ash.2025.10259)
Supplement: Moccia et al. supplementary material [file S2732494X25102593sup001.docx]

Supplementary Tables

Table A. **How easy or difficult have you found the new Millenium health informatics system to navigate? (N=11)**

| **Response option** | **n** | **%** |
| --- | --- | --- |
| Extremely difficult | 2 | 18.2 |
| Somewhat difficult | 8 | 72.7 |
| Neutral | 0 | 0.0 |
| Easy | 1 | 9.1 |

Table B. **Antibiotic indications are provided using a drop-down menu. How specific are the drop-down options for antibiotic indication?**

| **Response option** | **n** | **%** |
| --- | --- | --- |
| Not at all specific | 0 | 0.0 |
| Slightly specific | 3 | 27.3 |
| Moderately specific | 2 | 18.2 |
| Extremely specific | 1 | 9.1 |
| I have not used or am not aware of the drop-down options | 5 | 45.5 |

Table C. **Are the antibiotic indication and duration categories easily visible and quickly accessible?**

| **Response Option** | **n** | **%** |
| --- | --- | --- |
| Yes, both are easily visible and accessible | 2 | 20 |
| Only the indication is easily visible and accessible | 1 | 10 |
| Only the duration is easily visible and accessible | 2 | 20 |
| No, neither are easily visible or accessible | 5 | 50 |

Table D. **What do you find to be the most difficult about documenting antibiotic indication and duration using the new electronic health record system? (Check all that apply)**

| **Response Option** | **n** | **%** |
| --- | --- | --- |
| System navigation | 5 | 62.5 |
| Workflow-related barriers | 3 | 37.5 |
| Time consuming | 4 | 50 |
| Length of drop-down list | 0 | 0 |
| Was not aware of it | 2 | 25 |
| Have not been using it | 1 | 12.5 |
| None | 1 | 12.5 |

Table E. **Suggested improvements for documenting antibiotic indication and duration**

| **Suggested improvement** | **Number of respondents (n)** |
| --- | --- |
| Specific disease order sets | 1 |
| Reminder to do it | 1 |
| Drop-down menu | 1 |
| “Soft” popup notice (not a hard stop) with link to a clear page to pick/type indication | 1 |
| Fine as is | 1 |
| Pre-set options | 1 |
| Make it mandatory | 1 |
| More visible indication field | 1 |
| Check boxes | 1 |

Respondents were asked: *“What would make it easier for you to document an indication and a duration when ordering an antibiotic?”* Each participant could provide a single open-ended response.

Table F. **Do you think having order sets for specific disease processes would support your antibiotic prescribing practices to include indication and duration?**

| **Response Option** | **n** | **%** |
| --- | --- | --- |
| Strongly agree | 5 | 45 |
| Agree | 3 | 27 |
| Neutral | 1 | 9 |
| Disagree | 1 | 9 |
| Strongly disagree | 1 | 9 |
